# How does burnout relate to daily work-related rumination and well-being of psychotherapists? A daily diary study among psychotherapeutic practitioners

**DOI:** 10.3389/fpsyt.2022.1003171

**Published:** 2023-01-04

**Authors:** Katharina Gossmann, Regina Franziska Schmid, Carina Loos, Alessandra Barbara Anneliese Orthmann, Rita Rosner, Antonia Barke

**Affiliations:** ^1^Department of Psychology, Catholic University of Eichstätt-Ingolstadt, Eichstätt, Germany; ^2^Division of Clinical Psychology and Psychological Intervention, Institute for Psychology, University of Duisburg-Essen, Essen, Germany

**Keywords:** work-related rumination, ecological momentary assessment, psychotherapists, mental health, psychological well-being, burnout, daily diary

## Abstract

**Objective:**

This is the first study to use a daily diary design to investigate the relationship between daily work-related rumination (WRR), daily well-being, and burnout symptoms among psychotherapeutic practitioners.

**Method:**

In total, *N* = 58 psychotherapeutic practitioners participated in the study. For 4 weeks, the participants received a daily evening prompt on weekdays asking about their WRR and well-being. The burnout level of the psychotherapists was assessed using Maslach Burnout Inventory (MBI) prior to the daily diary period and afterward. The MBI measures the level of work-related distress on three subscales: emotional exhaustion (EE), depersonalization (DP), and personal achievement (PA). Two main analyses were performed: Based on the hierarchical structure of the data we performed random intercept and slopes models. These models examined the association between daily WRR and daily well-being, and the relationship between pre-burnout and daily WRR and daily mood. Secondly, linear regressions with the post-MBI subscales as criterion and the daily diary variables as predictors were calculated to assess their contribution to post-burnout.

**Results:**

The compliance rate in our study was 76.8%. Daily WRR and pre-assessment EE were associated with all aspects of reduced daily well-being: bad mood, increased nervousness, and tiredness after work. Daily tiredness and nervousness played a differential role in predicting post-burnout.

**Conclusion:**

Our results indicated that daily rumination and pre-EE were associated with reduced daily well-being. As we are the first to present a daily diary study among psychotherapists, we examined the feasibility of the daily diary design in particular and ecological momentary assessment (EMA) in general in this population. Compliance rates compared well with other EMA studies, indicating that EMAs were a feasible assessment option for psychotherapeutic practitioners.

## Introduction

Psychotherapists are at an elevated risk of burnout due to the specific emotional demands resulting from their work with patients with mental disorders (1–3). Burnout is defined by the three dimensions of emotional exhaustion (EE), depersonalization (DP), and personal achievement (PA) (4). EE—the most frequently reported symptom of burnout—describes the stress resulting from a lack of emotional and cognitive distance from work. DP constitutes an attempt to establish distance from patients by developing an indifferent and cynical attitude toward their uniqueness. PA stands for effectiveness and a low level of PA is indicative of burnout. PA can be impaired by EE, DP, and a lack of resources. Taken together, the dimensions describe a reaction to interpersonal or chronic emotional stressors like work overload or social conflicts. The burnout level is high when EE and DP are high and PA low (4).

Systematic reviews show prevalence rates of up to 55% for moderate to high burnout symptoms among psychotherapists and mental health professionals. According to the systematic review of Simionato and Simpson (1) 18.3–39.9% of psychotherapists and clinical psychologists reported high levels of EE and 11–26.3% low or moderate levels of DP. Psychotherapists also reported moderately to strongly reduced experience of PA with 15–29.6% being in the low range (1). When comparing the burnout level between psychotherapists and other professions, general practitioners described similar levels of EE (34.1%) and DP (29.0%), and low PA (21.5%) (5). Among general health care workers during the COVID-19 pandemic, EE was high in 37% and moderate in 45% of participants, DP was high in 18% and moderate among 49%, and PA moderate among 38% and high among 51% (6).

Burnout among psychotherapists is associated with poorer mental and physical health conditions such as increased depression, anxiety, sleep issues, alcohol consumption, and physical pain as well as reduced effectiveness in their therapeutic work (7, 8). Young age, limited work experience, being in training, high workload, little support, difficulties in one’s own work-life-balance, limited role clarity, lack of regular clinical supervision, and a reduced perception of self-efficacy have all been identified as risk factors for burnout (1, 9–11). In a meta-analysis the number of working hours and interaction with patients showing aggressive or threatening behavior during therapy emerged as the main factors associated with more EE and DP and reduced performance (12). So far, it is unclear how burnout relates to daily well-being and mood. Well-being can be understood in different facets like in the Multidimensional Mood Questionnaire as bad vs. good mood, tired vs. awake, or nervous vs. calm (13–15) or like in the Positive and Negative Affectivity Scale as work-related affective well-being with aspects like bored vs. enthusiastic, tiredness vs. vigor, anxiety vs. comfort, depression vs. pleasure, or angry vs. placid [PANAS (16)]. The advantages of the MDMQ in comparision to the PANAS is, e.g., the multidimensional conceptualization covering mood and activity level or the economical shortness of the questionnaire (17). Based on these different conceptualizations, well-being is comparable to moods and affective states which affect our behaving, thinking, and experiencing of everything we do (15). Affective well-being as conceived here is a dynamic state, susceptible to short-term changes of its facets; burnout as assessed with the MBI describes a state, that endures in a medium timeframe.

Another factor influencing burnout among psychotherapeutic practitioners is affective work-related rumination (WRR) (18, 19). WRR is characterized by repetitive and non-constructive, negative thinking patterns regarding work-related topics out of working time (18). Mohr et al. (20) describe cognitive irritation which is related to rumination as the inability to cognitively “switch off” from a topic. However, some researchers argue that rumination may also include positive problem-solving aspects (21–23). The inability to “switch off” during leisure time and rumination after work seem to be affected by high cognitive and emotional job demands, heavy work load and time pressure, low spatial work-home boundaries, limited support, and a low degree of control at work (24–27). Allwood et al. (18) found gender differences for rumination with women reporting more pondering and affective work-related rumination than men. In the case of a person prone to rumination, WRR is associated with low well-being and vigor, high emotional exhaustion and need for recovery, as well as sleep impairment (25, 28–33). Affective rumination, except its facet of problem-solving pondering, can further mediate the relationship between burnout and higher psychological morbidity (34), and the relationship between boredom or overload at work, and emotional exhaustion and disengagement 2 weeks later (35). This negative facet of rumination appears related to different emotional problems and may become psychopathologically relevant by increasing symptoms of depression, anxiety, or gambling (22, 36–38). For this reason, rumination is also focused on in the treatment of depression (39). The positive counterpart of negative affective rumination, psychological detachment, is closely linked to a higher degree of life satisfaction, less burnout, and greater personal flourishing and functionality (23). Since our study focusses on stress and well-being in psychotherapists, we investigate the negative aspects of rumination that are related to burnout and depression. Thus, we define rumination in the sense of negative, repetitive thinking comparable to worries which also describe repetitive, negative thoughts without a current solution (40, 41). Previous studies used trait-based measures of rumination tendency, which focus on the person’s self-appraisal of their general cognitive style. However, this may miss periods of increased rumination due to high job demand in persons would not normally describe their cognitive style as ruminative. We investigated the state distress caused by WRR on a within-person level.

Cognitive styles such as rumination about work-related topics seem to be associated with burnout. However, since most studies were based on correlative retrospective assessments of rumination and burnout, they were unable to establish whether persons with a high level of burnout overestimated their WRR retrospectively. Additionally, these studies focused on a between-person level using trait measures of rumination. Therefore, a longitudinal approach with a day-to-day perspective was needed that also took off-work recovery into account (42). A methodological approach that is particularly suited for such research is ecological momentary assessment (EMA) including also daily diary designs. In EMAs, participants report symptoms *in situ.* This immediate assessment resolves the biases of retrospective study designs such as memory effects or duration neglect. Additionally, EMAs often use repeated measurement which helps to cater for intrapersonal processes, to show symptom dynamics, and to allow context sensibility (43–45).

Considering these advantages of EMA, several studies with EMA designs already examined WRR and its potential correlates (46). However, there are no studies that examine WRR itself or the effect of WRR and burnout on daily psychological well-being among the population of psychotherapists. Psychotherapists work in a close interpersonal context with patients and might ruminate not about their work in general but about patients and the therapy sessions in specific. EMA studies in other health-related professions showed that chronic stress assessed in a baseline questionnaire went hand in hand with a daily assessed negative after-shift-mood among nurses (47). An EMA study using heart rate and skin conductance among forensic nurses demonstrated that the daily reported burnout symptoms after work were associated with daily assessed job stress and aggressive behavior of patients (48). Furthermore, another EMA study with 43 university students showed that daily assessed mind wandering was prospectively related to daily mood. Accordingly, daily mood was affected as negative emotions decreased and positive emotions increased when the reported mind wandering was experienced as mainly pleasant. The results also suggested that a dispositional trait for ruminative negative pondering exacerbates bad mood in daily life (49). Ruminative patterns assessed *via* daily EMA seem to indicate persisting symptoms of depression (50) and are related to negative affect (51). Additionally, increased negative and decreased positive affect seems to predict rumination at a later measurement point (52). Daily assessed work-related rumination was related to sleep impairment as it mediated the relationship between work-related tasks that remained unfinished before the beginning of a weekend and sleeping impairment (31). Additionally, the positive facet of rumination—problem-solving pondering—revealed to be associated with less sleeping impairment. Another EMA study on ruminative self-focus (two-item-scale), potential mindfulness interventions, and burnout among young adults demonstrated that although mindfulness instruction interventions were not related to changes in mood, they fostered calmness. Additionally, this study revealed that burnout was positively associated with ruminative self-focus among the sample (53). Furthermore, EMA studies using a cross-lagged design demonstrated that work stress increases ruminative thinking in the evening as well as sleep impairment. However, work stress was not associated with rumination at the weekends (32).

Thus, rumination has already been examined in several EMA studies with a within-person focus showing that a daily assessment is feasible (31, 54), able to distinguish between worrying and ruminating (55), and reliable regarding its relationship with biochemical measurements of cortisol (56, 57). These studies also demonstrate for certain samples (e.g., teachers) that not only actually present stressful events but even the rumination about these events increase physiological arousal (57). However, most studies cited focus on rumination in general and not work-related rumination in particular and above all not to the specific demands of work-related thought in a mental health orientated profession with daily patient interaction.

Despite the particularly high demands placed on psychotherapists and the increased burnout risk for them, little is known about contributing factors. Especially factors that may be targets for intervention, such as rumination about one’s therapies and patients, and the relationship of such rumination with daily well-being and burnout have never been examined in psychotherapists’ daily life, despite their potential of informing preventive measures. The present study aimed to increase knowledge about rumination, daily well-being, and burnout in a sample of psychotherapeutic practitioners. In order to overcome the well-known biases of retrospective self-reports, we used a daily diary design with daily reports of work-related rumination and psychological well-being. The series of within-person data will be used to elucidate the relationship of psychotherapists’ daily rumination and well-being with burnout. Therefore, the overall aim of our study is to demonstrate the dysfunctional circle between burnout, daily WRR and daily well-being among mental health professionals. Thus, we consider within-person effects for daily WRR and well-being as well as the effects of the pre-assessed burnout level on daily measures and the effects of the daily measures on post-burnout. We aim to reveal dysfunctional circles maintaining and fostering burnout and point out implications which may help to interrupt such cycles. Since this is the first daily diary study among psychotherapists, we also focused on the feasibility of the use of daily diaries among this population by examining the compliance rates for the daily prompts.

## Materials and methods

The study was conducted according to the Declaration of Helsinki (58) and approved by the University’s Internal Review Board in January 2021 (ethics approval number: 039-2020). All participants received information about the aims, procedure, and the General Data Protection Regulation before they gave their informed consent to participate.

### Procedure

We conducted this study as an ecological momentary assessment over the course of 4 weeks with pre- and post-assessments framing the daily diary period. The pre- and post-assessment was presented *via* the Qualtrics software (59). The daily diary schedule involved time-based monitoring with fixed-time sampling using the Android application movisensXS, version 1.4.8 (movisens GmbH, Karlsruhe, Germany, 2022). The data assessment was carried out from January to April 2021. We followed the reporting guidelines for EMA studies outlined by Trull and Ebner-Priemer when drafting this manuscript (44, 45).

Data were collected using pseudonyms. All participants generated an individual ID in the pre-assessment, which they entered at the beginning of the daily diary part and at the post-assessment. The daily assessment was conducted as a fixed-time sampling and took place on weekdays (Monday to Friday) at 8 p.m. over the course of 4 weeks. The daily diary period ended with the post-assessment. At the end of the post-assessment, the participants could request individualized feedback on their personal results regarding the different constructs such as burnout, daily mood, and rumination. Prior to receiving this individualized feedback, the participants were given additional information on data protection and privacy, and had to give their informed consent. All study parts were presented in German.

### Recruitment and participant flow

The participants were recruited through a nationwide online survey that took place in 2020 (60). At the end of this anonymous survey, participants were presented with an advertisement for the daily diary study and were linked to an independent survey and invited to provide contact information if they were interested in participating in the future diary study. In response to this advertisement, *n* = 40 psychotherapeutic practitioners indicated their interest in the daily diary study. Additionally, we contacted national and regional associations for psychotherapists in training (PiTs) and licensed psychotherapists (LPTs) in Germany who sent out our study information in their newsletters or by mail.

These recruitment strategies resulted in a list of *n* = 265 PiTs and LPTs interested in the study. All potential participants were sent detailed study information and the pre-assessment questionnaire by email. The inclusion criteria were working as a psychotherapist and performing psychotherapy on at least 4 days/week.

A total of *n* = 178 therapists completed the pre-assessment. Of these, *n* = 58 did not provide contact information or a pseudonymous participant code and thus did not receive an access code the second part of the study—the ambulatory daily assessment. From the *n* = 120 therapists who were invited to the daily diary assessment, *n* = 66 participated in the daily diary study part. Of these, *n* = 42 participated using their own mobile device and *n* = 24 participated using programmed devices from the university because their own devices were incompatible with the assessment app. After 4 weeks of daily assessments, all participants received the post-assessment which was completed by *n* = 45 participants. After excluding participants with incomplete datasets or less than two daily assessments, the final sample consisted of *N* = 58 participants for the analyses involving the pre-burnout and the daily measures and *N* = 44 participants who additionally participated in the post-assessment and thus, reported a post-burnout level. Almost all participants of the post-assessment for burnout (*n* = 43) requested individualized feedback.

To investigate whether therapists who—after the initial questionnaire—agreed to participate in the daily diary part and those did not, we compared the groups with independent *t*-tests. The groups did not differ with regard to the MBI (4, 61) facets of EE [*t*(178) = −0.74, *p* = 0.46], DP [*t*(176) = −0.67, *p* = 0.51], or PA [*t*(178) = −0.90, *p* = 0.37]. The level of burnout did not seem to play a systematic role in the therapists’ decision to participate in the daily diary or not.

### Measures

#### Pre- and post-assessment questionnaires

In the pre-assessment, participants reported demographical data like gender (female vs. male vs. diverse), age, training level (Psychotherapists in Training [PiTs] vs. Licensed Psychotherapists [LPTs]), time since start of licensed practice (LPTs)/beginning of training (PiTs), type of licensure (children and adolescents vs. adults), type of training institute (university vs. private), and the number of therapy sessions per week.

The burnout level of the participants was examined in the pre- and post-assessment using Maslach Burnout Inventory (4, 61). The MBI consists of three different scales: emotional exhaustion (EE—9 items), depersonalization (DP—5 items), and personal achievement (PA—8 items). In detail, the MBI scale for EE describes the exhaustion and frustration caused by a person’s own job requirements with items such as “I feel emotionally exhausted because of my work.” DP stands for the tendency to treat one’s patients like objects and to become emotionally numb (e.g., “I get the feeling that I treat some patients impersonally, as if they were objects.”). If a therapist feels successful and empathic while performing therapy, the level of PA is high (e.g., “I find it easy to build a relaxed atmosphere with my patients.”). Higher ratings for EE and DP in combination with lower ratings for PA indicated a higher degree of burnout (62). Participants rated their agreement with all items on a 7-point Likert scale ranging from “never” to “daily.” The levels of EE, DP, and PA can be classified to estimate the level of burnout: EE (scale range 0–54) with the level < 17 regarded as low, 18–29 as medium, and >30 as high; DP (scale range 0–30) with <5 regarded as low, 6–11 as medium, and >12 as high, and PA (scale range 0–48) with level < 33 regarded as low, 34–39 as medium, and >40 as high. In our assessment, item 24 of the DP subscale reduced the internal consistency of the subscale and thus was excluded from further analyses. The internal consistency (Cronbach’s Alpha and McDonald’s Omega) of the MBI scales in our sample was α = 0.92 (ω = 0.93) for EE, α = 0.70 (ω = 0.79) for DP, and α = 0.78 (ω = 0.78) for PA.

#### Daily diary

The daily diary part of the study consisted of items that assessed multiple aspects of daily perceptions about a person’s own therapeutic work, rumination, and well-being. Although, there are trait and state scales for rumination (21, 23, 31), these do not account for the specific work-related rumination related to ruminative thinking about one’s patients. Therefore, we established a one-item-EMA-scale assessing the distress caused be ruminative thoughts about one’s patients (“At this moment these thoughts about my patients and therapies are distressing for me”). The item was rated on visual analogue scales from 0 (not at all) to 10 (very much).

For daily well-being, we used a modified version of the Multidimensional Mood Questionnaire [MDMQ (13, 14)]—the standard measurement instrument in EMA studies with a good validity, sensitivity to change, and reliability (15). This questionnaire contained six adjective pairs (two on each of three scales: bad mood vs. good mood, tired vs. awake, nervous vs. calm). Each adjective pair is introduced with the statement “At this moment I feel…” and assessed using bipolar visual analogue scales (coded from 0 to 10): tired-awake, content-discontent, agitated-calm, full of energy-without energy, unwell-well, and relaxed-tense. For the analysis, all scales are coded in such a way that higher scores indicate the positive end of the scale (i.e., good mood, wakefulness, and calmness). Between-person and within-person reliabilities of the three MDMQ scales in our study were analyzed by applying generalizability theory (63). They were very high with *R*_*KF*_ = 0.99 (between) and *R*_*C*_ = 0.86 (within) for bad mood vs. good mood, *R*_*KF*_ = 0.98 (between) and *R*_*C*_ = 0.84 (within) for tired vs. awake, and *R*_*KF*_ = 1.00 (between) and *R*_*C*_ = 0.99 (within) for nervous vs. calm.

### Statistical analyses

Data were analyzed using SPSS statistics, version 28. First, the daily diary along with the demographical and pre- and post-assessment data were descriptively analyzed. In the next step, all person-related predictors (level 2) were grand-mean centered, and all daily diary predictors (level 1) were person-mean centered as recommended by Ohly et al. (64). Based on the hierarchical structure of the data—daily mood and rumination were nested in-person—we performed random intercept and slopes models. These models examined the association between daily rumination and daily mood (level 1), as well as the relationship between burnout (level 2) and daily rumination and daily mood. In addition, they looked at the interactions between burnout and rumination for daily mood. The three scales for daily mood (bad mood vs. good mood, tired vs. awake, nervous vs. calm), and burnout (EE, DP, PA) were analyzed separately. To examine potential interactions exploratively, we also included cross-level interactions between WRR and the MBI scales as predictors. All predictors and potential interactions were included simultaneously. These analyses were conducted using the burnout level in our pre-assessment.

Based on null models, we calculated the intraclass correlations (ICC) for all daily variables to estimate the variance between the intra-person (level 1) and inter-person (level 2) levels. To examine the model fit, we used chi-square tests to compare the differences in the log likelihood ratios and degrees of freedom between our null models and the final models.

To further investigate the potential influence of daily WRR and well-being on post-burnout, we performed additional multiple regression analyses with the three burnout facets (EE, DP, PA) as dependent variables with a subsample and the mean daily WRR and well-being as predictors.

## Results

### Descriptive statistics

#### Demographical sample characteristics

The final sample (*N* = 58) consisted of *n* = 34 LPTs (58.6%) and *n* = 24 PiTs (41.4%; including one psychologist working in psychotherapy not yet in training). Most participants were female (*n* = 50; 86.2%). In terms of type of licensure or training, the majority of the sample worked with adults (*n* = 45, 77.6%), and performed on average 17.7 therapy sessions per week (*SD* = 7.8). The mean age of the sample was 40.6 years (*SD* = 11.6), and the group of LPTs was significantly older than the PiTs [*t*(56) = 8.91, *p* < 0.001]. The LPTs had received their licensure an average 10.2 years previously (*SD* = 6.6) and the PiTs had been in training for an average of 3.5 years (*SD* = 3.9). Most of the PiTs were enrolled in private training institutes (*n* = 19, 82.6% of all PiTs), and just a small number (*n* = 4, 17.4% of all PiTs) in university-related training institutes. Tables 1, 2 give detailed information on the sample characteristics.

**TABLE 1:** Demographic information of participants.

Variable	*N* = 58					
	*n*	*M*	*SD*	*Mdn*	*Min*	*Max*
Age (years)	58	40.6	11.6	40	26	68
Time since licensure (years)—only LPTs	34	10.2	6.6	10	1	23
Time since beginning of training (years)—only PiTs	23	3.5	3.9	3	1	20
Therapy sessions per week	58	17.7	7.8	17.5	5	36

LPT, licensed psychotherapist; PiT, psychotherapist in training.

**TABLE 2:** Demographic information about the participants.

Variable	*N* = 58		Variable	*N* = 58	
	*n*	*%*		*n*	*%*
**Level of training**			**PiTs—Type of training institute (*n* = 23)**		
LPT	34	58.6	Private	19	82.6
PiT (+ *n* = 1 psychologist not yet in training)	24	41.4	University	4	17.4
**LPTs and PiTs—Type of license**			**Gender**		
Psychological psychotherapist (in training) for adults	45	77.6	Female	50	86.2
Psychotherapist (in training) for children and adolescents	10	17.2	Male	8	13.8
No specification	3	5.2			

LPT, licensed psychotherapist; PiT, psychotherapist in training.

#### Descriptive statistics for burnout and daily diary data

**FIGURE 1:**
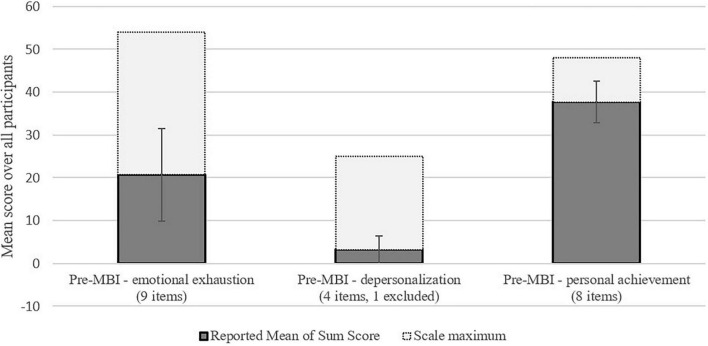
Mean scores of the component scales of the MBI. Error bars show standard deviations.

The burnout level at the pre-assessment of the sample based on the three MBI scales for EE was on average 20.7 ± 10.8 indicating a medium level, for DP on average 3.2 ± 3.3 indicating a very low level, and for PA on average 37.7 ± 4.8 in the medium range regarding the cut-offs (62). Figure 1 gives detailed information on the MBI subscales in our sample in comparison with the whole range of the scales.

Table 3 gives descriptive information about the daily assessed ratings. Daily well-being was rated in the dimensions bad vs. good mood (7.1 ± 1.9), tired vs. awake (5.1 ± 2.2), and nervous vs. calm (6.4 ± 2.0). For the one-item-scale for rumination after work (WRR), the sample reported a mean of 2.2 (±2.1).

**TABLE 3:** Descriptive statistics of all (component) scales.

Variable	*N* = 58				
	*M*	*SD*	*Mdn*	*Min*	*Max*
**Level-2—Pre-assessment**					
MBI—emotional exhaustion (9 items, range 0–54)	20.7	10.8	17	6	49
MBI—depersonalization (4 items, 1 excluded, range 0–24)	3.2	3.3	3	0	16
MBI—personal achievement (8 items, range 0–48)	37.7	4.8	38	19	48
**Level-1—Daily assessment**					
MDMQ—bad mood vs. good mood (range 0–10)	7.1	1.9	7.5	1	10
MDMQ—tired vs. awake (range 0–10)	5.1	2.2	5.0	0	10
MDMQ—nervous vs. calm (range 0–10)	6.4	2.0	7.0	1	10
Work-related rumination (range 0–10)	2.2	2.1	2.0	0	10
	***N* = 44**				
**Level-2—post-assessment**					
MBI—emotional exhaustion (9 items, range 0–54)	20.4	9.8	18	2	44
MBI—depersonalization (4 items, 1 excluded, range 0–24)	2.8	2.8	2	0	10
MBI—personal achievement (8 items, range 0–48)	38.4	5.4	39	19	48

MBI, Maslach burnout inventory; MDMQ, multidimensional mood questionnaire.

**FIGURE 2:**
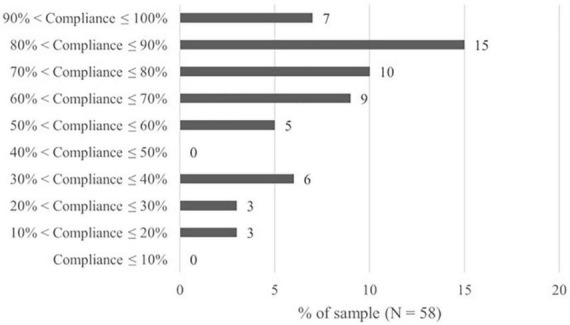
Distribution of compliance rates for the daily diary assessment.

Overall, each participant received 20 daily diary prompts over the course of the 4 weeks, resulting in 1,160 prompts across all included participants for the duration of the study. In the data analysis, we included all the participants who completed the daily questionnaire in response to at least two prompts. However, the lowest number of responded-to prompts was four and occurred three times; four participants completed all 20 notification forms (*M* = 15.8 ± 3.8, range 4–20). In total, the participating therapists responded to 810 prompts. Nine out of the 810 responses to prompts were incomplete. The response rate of the participants resulted in an overall compliance of 76.8% and ranged from 20% up to 100% with a high number of frequent responders (Figure 2). Individual compliance rates were significantly (but with a very small effect size) associated with the pre-assessed MBI scale for emotional exhaustion (*r* = −0.09, *p* = 0.007) indicating that participants who reported a higher level of EE responded to marginally fewer prompts. The individual response rate was not associated with demographic characteristics such as age, time since licensure or beginning of training, number of therapy sessions per week, gender, or level of training.

### Relationship between burnout, daily work-related rumination, and daily psychological well-being

The *ICCs* for all daily variables indicated that a considerable proportion of variance could be traced back to the within-person level: ρ = 0.44 for bad mood vs. good mood; ρ = 0.44 for tired vs. awake; ρ = 0.44 for nervous vs. calm; and ρ = 0.45 for rumination after work (WRR).

Our results indicated that there was a significant link between daily WRR and the psychological well-being of the therapists on the MDMQ-scales for *bad mood* vs. *good mood* [γ = −0.29, *t*(33) = −5.89, *p* < 0.001], *nervous vs. calm* [γ = −0.30, *t*(27) = −7.15, *p* < 0.001], and *tired* vs. *awake* [γ = −0.23, *t*(27) = −3.99, *p* < 0.001]. This indicated that a higher level of work-related rumination at the assessment time in the evening went hand in hand with lower mood, more nervousness and tiredness (Table 4).

**TABLE 4:** Multilevel models predicting daily level-1 variables for work-related rumination and the MDMQ scales.

	Bad mood vs. good mood		Tired vs. awake		Nervous vs. calm		Work-related rumination	
Fixed effects	Estimate (*SE*)	*p*	Estimate (*SE*)	*p*	Estimate (*SE*)	*p*	Estimate (*SE*)	*p*
Intercept	7.00 (0.15)	<0.001***	5.00 (0.18)	<0.001***	6.34 (0.15)	<0.001***	2.08 (0.19)	<0.001***
Pre-MBI—emotional exhaustion (EE)	−0.05 (0.02)	0.006**	−0.05 (0.02)	0.041*	−0.06 (0.02)	<0.001***	0.03 (0.02)	0.225
Pre-MBI—depersonalization (DP)	−0.02 (0.06)	0.717	0.02 (0.07)	0.735	−0.00 (0.06)	0.959	0.00 (0.03)	0.897
Pre-MBI—personal achievement (PA)	0.02 (0.03)	0.564	−0.01 (0.04)	0.797	0.00 (0.03)	0.905	0.05 (0.08)	0.543
Work-related rumination (WRR)	−0.29 (0.05)	<0.001***	−0.23 (0.06)	<0.001***	−0.30 (0.04)	<0.001***	–	–
Pre-MBI-EE × WRR	−0.01 (0.01)	0.344	−0.00 (0.01)	0.564	−0.00 (0.01)	0.557	–	–
Pre-MBI-DP × WRR	0.01 (0.02)	0.816	0.01 (0.03)	0.816	0.03 (0.02)	0.157	–	–
Pre-MBI-PA × WRR	0.01 (0.01)	0.377	0.01 (0.01)	0.307	0.01 (0.01)	0.524	–	–
**Random effect variances**	**Estimate (*SE*)**		**Estimate (*SE*)**		**Estimate (*SE*)**		**Estimate (*SE*)**	
Intercept	1.00 (0.24)***		1.45 (0.34)***		1.12 (0.26)***		1.31 (0.36)***	
Work-related rumination (WRR)	0.04 (0.02)		0.04 (0.03)		0.02 (0.02)		–	
**Model comparison**	**Null model**	**Main effects and interaction model**	**Null model**	**Main effects and interaction model**	**Null model**	**Main effects and interaction model**	**Null model**	**Main effects and interaction model**
–2* Log likelihood	3110.19	2434.13	3398.94	2698.31	3117.25	2403.60	2608.70	2132.96
χ^2^		676.06***		700.63***		713.65***		471.74***
*df*		8		8		8		3

*N*_1_ (persons) = 58, *N*_2_ (assessments) = 810.

***p < 0.001, **p < 0.01, *p < 0.05.

MBI, Maslach burnout inventory; MBI-EE, MBI-scale for emotional exhaustion; MBI-DP, MBI-scale for depersonalization; MBI-PA, MBI-scale personal achievement; WRR, work-related rumination after work (level 1); MDMQ, multidimensional mood questionnaire (level 1).

In terms of the burnout symptoms assessed prior to the daily diary, the MBI scale for EE was related to the daily psychological well-being on all three MDMQ component scales. Accordingly, higher scores for EE were associated with daily bad mood [γ = −0.05, *t*(58) = −2.83, *p* = 0.006], more tiredness [γ = −0.05, *t*(60) = −2.09, *p* = 0.041], and more nervousness [γ = −0.06, *t*(57) = −3.35, *p* < 0.001] in the therapists’ daily life. However, none of the pre-MBI scales was associated with daily WRR. We did not observe any interaction effects in our explorative interaction analyses.

To account not only for the influence of pre-assessed burnout on WRR and daily well-being but also for the influence of rumination and daily well-being on the burnout that was measured after the daily diary period, we conducted multiple regression analyses for all burnout scales in the post-assessment. For EE, the overall model [*F*_(4,704)_ = 67.27, *p* < 0.001] explained 27% of variance (*R^2^_*Adjusted*_* = 0.27) and showed a high effect size of Cohen’s *f*^2^ = 0.38. The detailed analysis revealed two significant predictors: daily tiredness (β = −0.18, *p* < 0.001) and nervousness (β = −0.41, *p* < 0.001). For DP, neither total model nor the predictors proved significant [*F*_(4,704)_ = 1.40, *p* = 0.252]. The final regression with the criterion PA also did not yielded a significant model and no predictors proved significant [*F*_(4,704)_ = 1.56, *p* = 0.185] (Table 5).

**TABLE 5:** Regression models for burnout (emotional exhaustion, depersonalization, personal achievement) based on daily rumination and well-being (good vs. bad mood, tired vs. awake, nervous vs. calm).

	Estimate (*SE*)	Standardized beta	*p*
**MBI—Emotional exhaustion (EE)**			
Intercept	47.70 (2.88)		<0.001
MDMQ—bad vs. good mood	−0.06 (0.50)	−0.01	0.906
MDMQ—tired vs. awake	−1.37 (0.32)	−0.18	<0.001
MDMQ—nervous vs. calm	−3.04 (0.37)	−0.41	<0.001
Work-related rumination (WRR)	−0.20 (0.31)	−0.03	0.516
**MBI**—**Depersonalization (DP)**			
Intercept	7.68 (3.76)		0.048
MDMQ—bad vs. good mood	−0.66 (0.64)	−0.27	0.307
MDMQ—tired vs. awake	−0.22 (0.43)	−0.10	0.613
MDMQ—nervous vs. calm	0.10 (0.50)	0.05	0.846
Work-related rumination (WRR)	0.13 (0.40)	0.06	0.747
**MBI—Personal Achievement (PA)**			
Intercept	35.14 (1.87)		<0.001
MDMQ—bad vs. good mood	0.20 (0.32)	0.04	0.533
MDMQ—tired vs. awake	0.35 (0.21)	0.08	0.094
MDMQ—nervous vs. calm	−0.05 (0.24)	−0.01	0.840
Work-related rumination (WRR)	0.17 (0.20)	0.04	0.397

Model: *MBI-EE—F*_(4,704)_ = *67.27, p* < 0.001, adjusted *R*^2^ = 0.27. *MBI-DP—F*_(4,704)_ = 1.40, *p* = 0.252, adjusted *R*^2^ = 0.03. *MBI-PA—F*_(4,704)_ = 1.56, *p* = 0.185, adjusted *R*^2^ = 0.00. MBI, Maslach burnout inventory; MBI-EE, MBI-scale for emotional exhaustion; MBI-DP, MBI-scale for depersonalization; MBI-PA, MBI-scale personal achievement; WRR, daily work-related rumination after work; MDMQ, multidimensional mood questionnaire (assessed daily).

## Discussion

We conducted the first daily diary study among psychotherapeutic practitioners and reported results on the relationships between work-related rumination, daily well-being, and burnout. In general, the participating psychotherapists reported a medium level of EE, a low level of DP and high levels of PA, suggesting they were somewhat less burdened than previous research observed (1, 10, 61, 65).

Our study results showed that overall WRR was low in our sample regarding the scale maximum. Still, rumination was related to bad mood, more tiredness, and more nervousness after work. Pre-assessed emotional exhaustion—as an aspect of burnout among psychotherapists—was significantly associated with bad daily mood, tiredness, and nervousness. However, pre-assessed burnout symptoms were not directly associated with daily work-related rumination. To take time effects into account, we also tested whether daily well-being and WRR related to burnout in our post-assessment. The MDMQ-scales for tiredness and nervousness were associated differentially with EE at the post-assessment. The compliance rate (76.8%) among the participating psychotherapists demonstrated that daily diary and EMA research designs were a feasible option for psychotherapeutic practitioners.

### Relationship between daily work-related rumination, well-being, and burnout

In cross-sectional studies, psychotherapeutic practitioners showed a high risk of burnout (1–4, 10, 65). Burnout was associated with different risk factors and, among other things, with affective work-related rumination (18, 19, 53). WRR relates to different health outcomes like decreased well-being and high EE (25, 28–31, 33). First EMA studies demonstrated that daily well-being was associated not only with WRR but also with burnout (47–49, 51, 52), and burnout is positively associated with a ruminative self-focus (53).

In our study, daily WRR after work was associated with bad mood, tiredness and nervousness. EE at pre-assessment was related to better daily well-being, such as good mood, feeling more wakeful and calmer. The other burnout scales showed no effects. Based on our results, we concluded that daily rumination after work was linked to the reduced well-being of psychotherapists. Therefore, our study did not only consider within-person differences by analyzing the effects of daily WRR on daily well-being, but also included cross-level analyses by considering the potential effects of burnout and the interaction between burnout and daily WRR on daily well-being.

The main effect of WRR on daily well-being observed in the current daily diary study was in line with previous cross-sectional research indicating that negative rumination patterns after work and difficulty switching-off from work could negatively affect well-being and impair mental health (47–49). Our results for WRR also go along with previous EMA research showing that rumination in general goes along with negative affect (51). We demonstrated that this effect of daily rumination also became clear in the case of psychotherapists in a longitudinal study. Furthermore, we concluded that well-being assessed in a daily setting was associated with burnout and, more particularly, with EE. This expanded our knowledge about these relationships since, in contrast to retrospective cross-sectional designs, we were able to rule out retrospective reporting bias as the reason for the correlation. However, we did not find a direct association between pre-assessed burnout and WRR, indicating that pre-existing EE as well as other MBI facets did not appear to lead to more daily WRR *per se*. Thus, our study does not support previous results regarding the positive relationship between burnout and rumination (53). Although, it needs to be considered that the study of Huffziger et al. (53) did not assess work-related rumination and examined another sample—young university students. In our study daily rumination was associated with daily well-being, and, as the regression with the assessment at the end of the daily diary showed, with future burnout facets (see below for a detailed discussion). Prevention strategies for burnout and daily well-being among psychotherapists may need to focus on daily WRR, too. To decrease daily WRR and potentially increase daily well-being, detachment strategies for psychotherapists could be addressed in prevention programs or during psychotherapist training. Previous studies already demonstrated that mindfulness-based interventions among recurrently depressed patients were able to reduce negative rumination (50) and thus could also be a promising approach for the prevention of rumination among psychotherapists. Cognitive control plays seem also reduce negative emotion regulation including rumination in the daily life (66). Therefore, decreasing rumination in general, could be one piece in prevention and intervention strategies to prevent or reduce job-related emotional problems among psychotherapists, as a high level of rumination can become problematic and is related to psychopathologically relevant symptoms, such as anxiety and depressive symptoms (36, 37). Additionally, future research should include also daily measures of well-being and WRR at the weekends to enable cross-lagged analyses like in the study of Vahle-Hinz et al. (32). These studies could reveal time effects of rumination and therapeutic experiences on daily well-being.

As we were also interested in how the WRR and well-being during the EMA period was related with the burnout level after the end of the assessment, we conducted additional regression analyses. More daily tiredness and nervousness was associated with more EE at the end of the assessment. These results indicate that daily well-being may contribute to the development of burnout in general and EE in particular.

However, as our sample in the regression analyses consisted of only 44 participants, further research should examine whether these results prove robust and investigate the influence of daily WRR on burnout considering different positive and negative facets of work-related thinking by in further and larger samples.

### Feasibility of daily diary designs for studies with psychotherapeutic practitioners

Our study achieved a compliance rate of 76.8% among psychotherapists, and very few participants had to be excluded because they responded to fewer than two prompts. According to the meta-analysis of Wrzus and Neubauer (67), the compliance rate of 76.8% was on a high level comparable to other EMA in a range of research field, that showed an average compliance rate of 79.2%. Daily diary and EMA designs in general seemed to be a feasible option for psychotherapeutic practitioners. However, the daily diary requirements may have deterred a few potential participants from the outset: although 178 therapists participated in the pre-questionnaire, only 66 agreed to participate in the second study part by completing the daily questionnaires. This meant that only around one third of all pre-questionnaire participants were willing to provide daily reports, with a further third of this smaller group dropping out before the post questionnaire. Informal feedback received by mail indicated that this may not be solely attributable to the demands of the daily diary, but rather that many motivated PiTs would not reach the number of 4 days with therapies stipulated as inclusion criterion.

For the interpretation of the current study results, it should be borne in mind that we found no significant difference regarding the burnout level between participants of the daily diary study and those who answered only the pre-assessment. Also, demographical characteristics were not associated with the participation in the daily diary study part or the individual compliance rates during the daily diary assessment. Nevertheless, we found differences regarding the compliance rates in relation to the pre-assessed burnout level. These findings indicate that participants with higher EE answered fewer daily prompts. When interpreting this correlation, it should be taken into account that a correlation coefficient of *r* = −0.09 is very small.

### Limitations

The recruitment strategy addressed interested psychotherapeutic practitioners in Germany. As the inclusion criteria required that the participants were in contact with patients at least 4 days per week, it is possible that psychotherapists working part-time or psychotherapists in training with a lower number of working hours were systematically excluded from the study. This again constituted a potential recruiting bias. Another restriction concerning our sample was that the daily diary design included fixed-time sampling. This explains why participants who knew that they were not available at the specific daily assessment time, might have decided against participating. As the inclusion criteria, along with the assessment time and period, were provided in the study information, it is probable that self-selection took place.

Due to the nature of the study, we were not able to include all potentially relevant covariates for burnout, daily well-being, and rumination. Relevant covariates may have been, e.g., the therapeutic orientation—which we asked for, but could not include in the analyses due to the small group sizes of the orientations—, the number or therapy sessions per week, case supervision, personal analyses, or specific populations predominantly treated such as patients with complex needs. These covariates could potentially also influence the daily well-being, and the level of WRR or burnout among psychotherapists. As we were only interested in work-related rumination directly on days when therapy was performed, our daily assessments only took place on weekdays. Consequently, we cannot provide information on general rumination tendencies or rumination on weekends. Thus, we also did not conduct cross-lagged analyses as our study was not designed for time lagged processes. Further studies should therefore include assessment on weekends like Vahle-Hinz et al. (32) to enable analyses over time considering cross-lagged effects also on weekends.

For the assessment of daily well-being, we used a well-established and validated measure (15). To assess WRR in our daily diary design, we established a new one-item scale for WRR related to one’s patients. However, in the meantime Hoebeke et al. (68) developed a five-item general rumination questionnaire with good psychometric properties for EMA studies. This questionnaire could be adapted to work-related rumination, validated among different professions, and adjusted to professions in the health sector with patient interaction. Additionally, further EMA-research on rumination should consider more extensive scales for WRR like Syrek et al. (31) did and adapt these for patient-related professions. These scales also should cover different positive and negative facets of rumination as previous research demonstrated the interdependence of positive, negative, and neutral rumination as well as different correlates with these facets (21, 23, 46).

Further limitations refer to the sample characteristics: Our sample consists of mainly female psychotherapeutic practitioners. Although this distribution of gender reflects the demographical epidemiological distribution of gender among psychotherapists in Germany (69), the generalizability of our results to male psychotherapist in general may be limited.

## Conclusion

Daily diary studies and consequently EMA designs are a feasible option for psychotherapists and should be used more frequently to assess the factors that influence work-related rumination on well-being among psychotherapeutic practitioners. This study built on the knowledge regarding the mechanisms that may contribute to diminished well-being and burnout in psychotherapists. Work-related rumination and pre-assessed burnout, especially emotional exhaustion, appeared to be potential risk factors for reduced daily well-being. Additionally, different aspects of daily well-being are related to the burnout level at the post-assessment. These findings have implications for future prevention and intervention efforts, which should focus on the ability of psychotherapists to switch-off from work, and foster strategies to achieve detachment from patient and therapy-related issues during psychotherapists’ leisure time.

## Data availability statement

The raw data supporting the conclusions of this article will be made available by the authors, without undue reservation.

## Ethics statement

The studies involving human participants were reviewed and approved by the Institutional Review Board (IRB) of the Catholic University of Eichstaett-Ingolstadt in January 2021 (ethics approval number: 039-2020). The patients/participants provided their written informed consent to participate in this study.

## Author contributions

KG designed the study, recruited the participants, gathered the data, drafted the manuscript, and carried out the statistical analysis. AO and CL were involved in gathering the data. AB and RR supervised the study and revised the manuscript. AB and RS were involved in designing the study, analyzing the data, and in drafting the manuscript. All authors read and approved the manuscript and its final version.
